# ^1^H NMR-Based Analysis to Determine the Metabolomics Profile of *Solanum nigrum* L. (Black Nightshade) Grown in Greenhouse Versus Open-Field Conditions

**DOI:** 10.3390/metabo15050344

**Published:** 2025-05-21

**Authors:** Lufuno Ethel Nemadodzi, Gudani Millicent Managa, Ndivho Nemukondeni

**Affiliations:** 1 Department of Agriculture and Animal Health, University of South Africa, Johannesburg 1709, South Africa; 2 Department of Animal Sciences, Tshwane University of Technology, Pretoria 0001, South Africa

**Keywords:** *Solanum nigrum*, growing conditions, metabolites profiling, glycocholate, 3-chlorotyrosine, chlorogenate, caffeine

## Abstract

Background: Equally with other indigenous green leafy vegetables, *Solunum nigrum* L. has been widely consumed by the VhaVenda tribe found in the Limpopo Province of South Africa since ancient times as a source of food diversification due to its higher-quality nutritional value, sustainability, food security, and medicinal benefits. It is mostly cultivated from seeds in seedling trays and transplanted in the open field, and at the maturity stage, marketing and distribution are mainly conducting through informal markets (i.e., street vendors). However, recently, it can be found in selected supermarkets and commercial grocery stores in South Africa. The leaves and young shoots of *S. nigrum* are cooked solely and/or as a supplementary vegetable with *Brassica rapa* L. subsp. *chinensis* (Chinese cabbage), *Spinacia oleracea* L. (spinach), *Amaranthus graecizans* L. (green amaranth), *Solanum lycopersicum* L. (tomato), and/or cooking oil for flavor. Objective: Contrary to other green leafy vegetables, few studies have been conducted on the metabolites released by *S. nigrum* and the influence of growing conditions on the metabolites thereof. Method: A ^1^H-nuclear magnetic resonance tool was used to identify the untargeted metabolites released by *S. nigrum*, and spectra were phase-corrected and binned with MestReNova and statistically analyzed with SIMCA 18.0.2. Results: The findings showed that a total of 12 metabolites were detected between the growing conditions. Eleven similar metabolites, such as glycocholate, chlorogenate (human health benefits), caffeine for its bitter taste, choline, 3-Chlorotyrosine (antidiabetic, blood pressure), etc., and a few vital soluble sugars, were detected in *S. nigrum* samples grown in the open field and greenhouse-cultivated. Glucose was exclusively detected in the *S. nigrum* grown under greenhouse conditions.

## 1. Introduction

Since ancient times, indigenous green leafy vegetables (IGLVs) such as *Amaranthus* spp. (Vowa); *Corchorus olitorius* L., commonly known as Mallow jute (Delele); *Cloeme monophylla* L., also referred to Spindle pod (Mutohotoho); *Bidens pilosa* L. (Blackjack), indigenously referred to as “Mushidzhi”; *Brassica rapa* L. *subsp. chinensis* (Chinese cabbage), also known as Mutshahina; and *Solanum nigrum* L. (Muxe), among others, have always played a vital role in the traditional diet in the rural and peri-urban communities of South Africa, mainly among the VhaVenda tribe of the Limpopo Province. These vegetables are consumed either cooked or in a dried form with a staple food made of ground *Zea mays* L. (maize) and/or *Sorghum bicolor* L. (sorghum millet), known as pap or porridge, which is commonly and tribally referred to as “Vhuswa”. Indigenous green leafy vegetables play a vital role in combatting hunger, ensuring food security, and alleviating malnutrition, and they are mostly suitable for dietary intervention programs [1]. They are also rich in nutraceuticals that provide scientific justification and possible mechanisms for their use in the management of a wide range of ailments, which include diet-related, non-communicable diseases such as diabetes, hypertension, and cardiovascular diseases, as reported by Moussa et al. [2]. Several studies have indicated that African leafy vegetables (ALVs) contain micronutrient levels as high as, or even higher than, those found in most exotic leafy vegetables (LVs) [3,4,5,6,7,8]. However, false pre-conceived notions and stigmas attached to indigenous vegetables, as well as their association with poverty and primitiveness, have led to less consumption and non-preferability among the youth, high-income individuals, and urban households. According to Van Averbeke et al. [9], poor rural households consume these leafy vegetables (LVs) more than their wealthier counterparts. This has intensively contributed to South Africans overlooking/disregarding the numerous known and unknown nutritional and medical benefits possessed by leafy vegetables.

According to Calumpang et al. [10], Solanaceae (nightshade family) is an agronomically and botanically diverse plant taxonomic group, with members ranging from vegetable crops to medicinal plants and ornamentals. A few representative crops of economic importance include *S. lycopersicum* [11,12], *Solanum melongena* L. (eggplant) [13], and *Capsicum annuum* L. (pepper) [14,15]. *Solanum nigrum* (bitter-green leafy vegetable species), used in the current study, is an annual plant growing to almost 75 cm in height [16]; it has a simple leaf morphology, alternate margins with blunt teeth, and is slightly hairy. In Southern Africa, subsistence farmers cultivate nightshade vegetables on a small scale and market them to generate income and improve their livelihoods [1]. Azeez et al. [17] revealed that *S. nigrum* plants do not need extensive fertilizer application, thrive in drought, and are less prone to pest attack; therefore, they are cost-effective and environmentally friendly compared to commercial leafy vegetables. *Solanum nigrum* has been planted and used primarily for human consumption, as a medicinal plant, and to a lesser extent, as a livestock feed or supplement, as reported by Sangija et al. [18]. In addition, research on nightshade, which includes nutritional composition and/or elements [8,19], chlorophyll [20,21], and antioxidant and phenolic compounds [22], has been conducted. Furthermore, detailed metabolomic studies on *S. nigrum* berries [10] have been undertaken and are report their high nutritional potential and ability to curb hunger [23]. These berries may be a good feed supplement to livestock due to their rich nutritional content, containing vitamin A, vitamin C, calcium, and iron, and might help reduce feed costs. However, there is a huge gap identified in the literature on the metabolomics profile on the leaves of nightshade, which makes the current study, to our knowledge, the first to report the metabolites identified in the leaves of *S. nigrum* using ^1^H nuclear magnetic resonance (NMR).

Recently, in South Africa, there has been a shift in scientific research which has led to the subsequent development of advanced analytical tools such as metabolomics. Metabolite profiling is a fast-growing technology useful for phenotyping and diagnostic analyses of plants. It is a key tool in the functional annotation of genes and the understanding of the cellular response to biological conditions, as described by Schauer and Fernie, [24]. According to Nemadodzi et al. [25], metabolomics has been widely applied in several human and plant studies and is gradually applied in soil science research. Despite the large number of metabolites yet to be identified, metabolomics has contributed significantly to furthering our comprehension of plant physiology and biology through the analysis of small chemical molecules that represent the end point of biological activities. Moreover, in recent decades, attempts have been made to improve plant behavior under both normal and stressed conditions [26].

Plants’ ability to chemically modify the core structures of specialized metabolites is the main reason the plant kingdom contains wide and richly diverse compounds [27]. The diversity of plant metabolites and the likely complicated regulatory mechanism highlight the necessity of exploring their underlying biochemical nature, as indicated by Hall et al. [28]. Metabolomics plays an increasingly important role in plant science, being aimed at the comprehensive analysis of the plant metabolome, which consists of primary and secondary metabolites [29]. However, due to their being few studies conducted using advanced metabolomic tools on indigenous green leafy vegetables such as *Amaranthus* spp. (amaranth) [7,30], *Chlorophytum comosum* L. (known as spider plant (Murudi)) [31], *C*. *olitorius* [32], and *Cucurbita moschata* Duch. (pumpkin leaves) or Thanga in TshiVenda [33], there is limited scientific literature on their biological constituents and the functions thereof. In addition to NMR, liquid chromatography–mass spectrometry (LC-MS) analysis is popularly used to determine the metabolite profile of various samples, as reported by Nemadodzi et al. [25]. Recently, scientists have gravitated towards gas chromatography–mass spectrometry (GC-MS) and headspace techniques in plant studies.

A study by Kirigia [34], conducted under greenhouse conditions, reported that secondary metabolites, such as the total phenolics (gallic acid equivalents), catechin equivalent flavonoids, and trolox equivalent antioxidants (TEA), were THE highest in concentration in *Solanum scabrum* mill (African nightshade) 90 after planting. In another study, a higher level of lutein was reported in *S*. *scabrum*, whilst glucosinolates were not found [35]. El-seedi et al. [36] found caffeic and sinapic acids in *S*. *lycopersicum* and *S*. *melongena*.

The advantages of using NMR include its non-destructiveness, unbiasedness, and quantitative, uncomplicated sample preparation, and it may be used to study compounds that are difficult to detect using gas and chromatography–mass spectrometry (GC-MS) or LC-MS [25]. In addition, NMR has been proven to detect targeted and untargeted metabolites in a sample. The initial hypothesis of the current study was that different growth conditions (open field versus greenhouse-grown) will influence the release of the different metabolites. The current study aims to evaluate the metabolites released by *S. nigrum* grown in open-field and greenhouse conditions.

## 2. Materials and Methods

Greenhouse cultivation conditions: The pot experiment was conducted in a greenhouse with a minimum and maximum air temperature range of 7.4 and 44.9 °C, situated at the University of South Africa, Florida Science Campus, Roodepoort (latitude: −26°9′29.274″; longitude: 27°55′17.663″). The average relative humidity inside the greenhouse was recorded as 68% [37] during the planting period (October 2021 to February 2022). The plastic pots (18 cm diameter, 14.5 cm height, and 18 cm width) were used in growing seeds. The growth cycle lasted 3 months in the summer seasons of October to December 2021 for both cropping systems.

Open-field cropping system: The experiment was conducted in Itsani village, situated about 6 km southwest of Thohoyandou in the Thulamela Local Municipality, Vhembe District, Limpopo Province of South Africa. Itsani village has a latitude of −22.94786, a longitude of 30.47276, and an altitude of approximately 1140 m above sea level. The planting took place during the summer period (October to March), and temperatures ranged from 26 to 32 °C with an average rainfall of ±500 mm [7].

Open-field agronomic preparations: The soils were prepared using a hand hoe, and seedlings were planted with a spacing of 30 cm between the plants. Watering was conducted once a day in the morning, and no fertilizers were applied.

**Figure 1:**
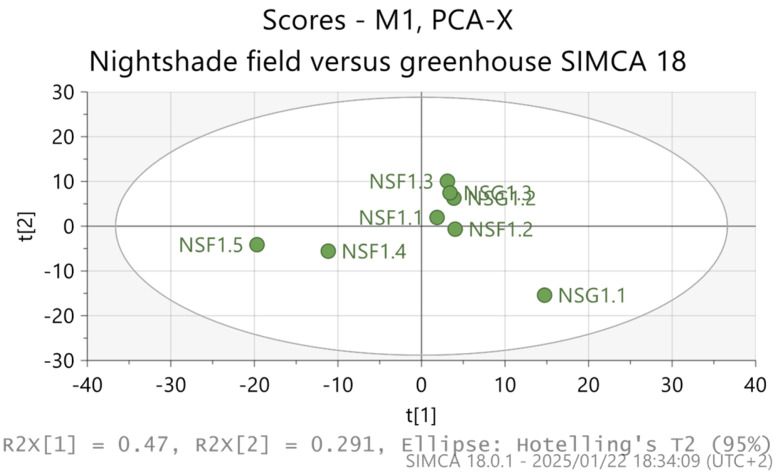
PCA (unsupervised) model showing the scattered samples collected from field-grown (NSF) and greenhouse-cultivated (NSG) *S. nigrum*. t[1] and t[2] represent the first two principal data components.

**Figure 2:**
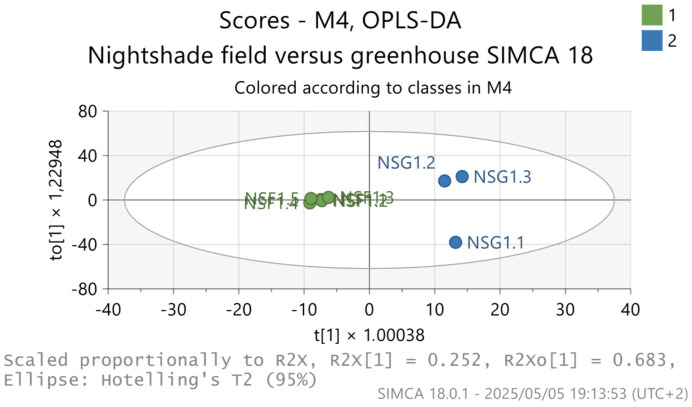
OPLS-DA model showing a distinct separation between *S. nigrum* samples collected from the open field (NSF) versus those collected from the greenhouse (NSG). t[1] and t[2] represent the first two principal data components.

Leaf collection: Individual selected fresh leaves of *S. nigrum* grown in the open field, coded as NSF, as well as those of *S. nigrum* cultivated in the greenhouse, coded as NSG (see Figure 1 and Figure 2), were harvested at the 8-leaf stage in the summer period of December 2021, dried at room temperature at 27 °C, and stored until use.

Leaf sample preparation: Dried leaves from each cropping system were grounded into a fine powder with Russel Hobbs Blender (500 W, Zhongshan, China) to produce a homogeneous powdered samples and were used for NMR metabolomics analysis.

NMR metabolomics analysis: For NMR analysis, deuterated methanol (CD_3_OD), KH_2_PO_4_, sodium deuterium oxide (NaOD), trimethylsilyl propionic acid sodium salt (TSP), and deuterium oxide (D_2_O) was supplied by Sigma-Aldrich (Darmstadt, Germany). Buffer was prepared by adding 1.232 g KH_2_PO_4_ to 100 mL of D2O, with 10 mg TSP (0.1%) added as a reference standard. The pH of the solution was adjusted to 6. The protocol reported by Kim et al. [38] was adapted for the extraction procedures, with a few adjustments. A dried leaf sample of 50 mg was transferred to 2 mL Eppendorf tubes and extracted with 750 µL of deuterated methanol and 750 µL KH_2_PO_4_ buffer in D_2_O containing 0.1% TSP. The Eppendorf tubes were vortexed for 1 min at room temperature and ultra-sonicated for 20 min without heating. The solutions were centrifuged for another 15 min at 10,000 rpm to separate the supernatant from the precipitates. The supernatant was transferred to standard 5 mm NMR tubes and subjected to ^1^H NMR analysis.

The ^1^H NMR measurements were performed on a Varian 600 MHz spectrometer (Varian Inc., Palo Alto, CA, USA). The acquisition time of each ^1^H NMR spectrum was 7 min, which consisted of 32 scans with a sweep width of 20 ppm. Gradient shimming was used to improve the homogeneity of the magnetic field, and each sample was replicated five times. All spectra were phase-corrected and binned with a bucket size of 0.04 ppm to 10.00 ppm using MestReNova, as prescribed by Maree and Viljoen [39], before being statistically analyzed with SIMCA 18.0.2 (Umetrics, Umea, Sweden). Data scaling was conducted using the Pareto method. Unsupervised principal component analysis (PCA) and supervised orthogonal partial least squares discriminant analysis (OPLS-DA) models were used to illustrate the distinctive separation between the two sampling sites where leaves were collected [40,41]. The annotation, detection, and identification of non-targeted metabolites was carried out using Chenomx NMR suite 11.0 (Edmonton, AB, Canada), which has specific characteristics (also known as values) for a variety of metabolites [42]. The Chenomx library manager was used to obtain the compounds database, while Chenomx compound builder version 11 was used to create 1D NMR chemical structures with the cluster/number of peaks, shapes, locations, and NMR signal regions at parts per million (ppm).

## 3. Results

The findings of this study showed that the PCA displayed no differences in the samples from both growing environments, as shown in Figure 1. The R^2^ and Q^2^ as well as their components derived from PCA and OPLS-DA are indicated in Table 1 and Table 2, respectively. 

On the contrary, the unsupervised model showed a distinct separation of metabolites released by *S. nigrum* grown in the open field (green color) compared to greenhouse (blue color)-cultivated *S. nigrum*, as seen in Figure 2.

**Figure 3:**
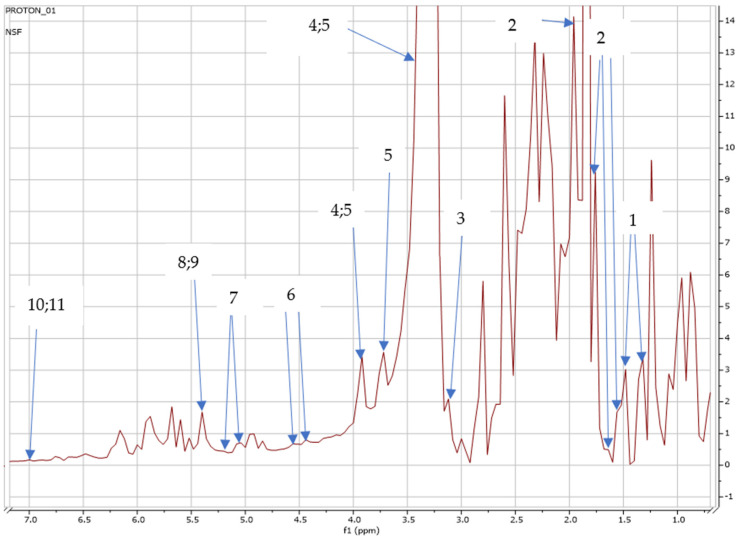
NMR full spectra showing peaks detected on *S. nigrum* cultivated in the open field: (1) alanine (1.45; 1.46 ppm); (2) glycocholate (1.5; 1.6; 1.7; 1.8 ppm); (3) choline (3.2 ppm); (4) betaine (3.3; 3.9 ppm); (5) caffeine (3.3; 3.5; 3.9 ppm); (6) galactose (4.6 ppm); (7) trehalose (5.2 ppm); (8) maltose (5.4 ppm); (9) sucrose (5.4 ppm); (10) chlorogenate (7.0 ppm); (11) 3-chlorotyrosine (7.0 ppm).

To detect and identify the secondary metabolites of field-grown *S. nigrum*, the NMR spectra of the current study were developed using Mestrenova Research Laboratory Chemistry, showing different peaks in the NMR ppm regions, as indicated in Figure 3.

The peaks and NMR regions (ppm) at which the metabolites were detected are shown in Table 3.

Additionally, external reference databases, namely, as Chenomx and the human metabolome data base (HMDB), were used to compare and confirm the NMR-specific characteristics [43] detected in the current study, as shown in Table 3.

**Figure 4:**
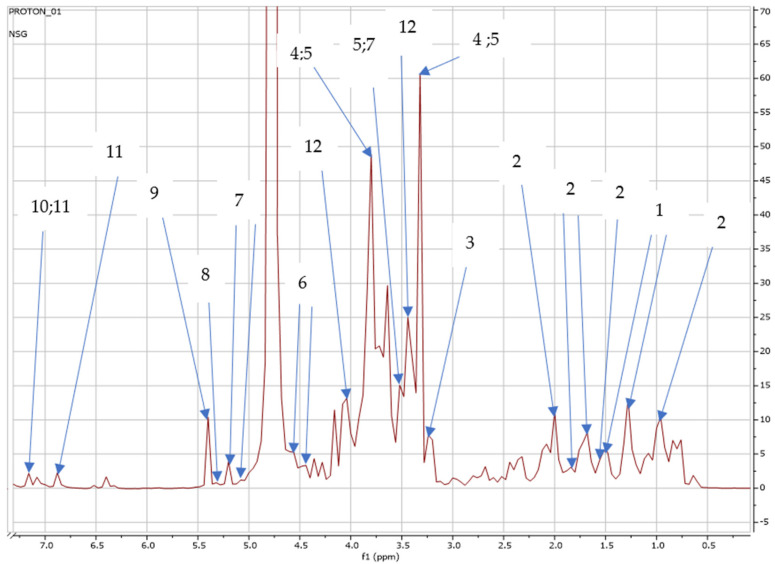
NMR full spectra showing peaks detected on *S*. *nigrum* cultivated in the greenhouse: (1) alanine (1.45; 1.46 ppm); (2) glycocholate (1.0; 1.5; 1.6; 1.7; 1.8 ppm); (3) choline (3.2 ppm); (4) betaine (3.3; 3.9 ppm); (5) caffeine (3.3; 3.5; 3.9 ppm); (6) galactose (4.6 ppm); (7) trehalose (3.5, 5.2 ppm); (8) maltose (5.4 ppm); (9) sucrose (5.4 ppm); (10) chlorogenate (7.1 ppm); (11) 3-chlorotyrosine (7.0; 7.1 ppm); (12) glucose (3.4, 4.0 ppm).

Interestingly, *S. nigrum* grown in the open field released the same metabolites as those cultivated in the greenhouse, with the exception of glucose, which was only found in the *S*. *nigrum* plants grown in the greenhouse, as shown in Figure 4 and Table 4.

## 4. Discussion

African indigenous leafy vegetables (AILVs) are key to establishing an affordable and sustainable solution to the threat of hunger and malnutrition in Sub-Saharan Africa [46,47,48]. Recent studies by Fan et al. and Lou et al. [49,50] reported that plants synthesize hundreds and thousands of structurally diverse and low-mass molecules known as specialized secondary metabolites, which provide humans with medicines, food additives, and natural insecticides. According to Fang et al. [51], a huge array of metabolites are produced by plants, far more than those produced by most organisms. Since plants are capable of designing and producing multifarious chemical compounds that serve mankind as foods and medicines, the effective engineering of the metabolic pathways in plants via modern biotechnology will bring more benefits to human beings [26]. According to Yamaki, Jia et al. [52,53], apart from the traditional functions of sugars in plants as metabolic resources for energy and carbon skeleton construction, sugars could also be involved in other functions such as seed germination, vegetative and reproductive growth, and senescence. Sugars are not only important components of fruit flavor and nutritional composition; they also play a vital signaling role during plant growth and are involved in regulating gene expression during plant development [54,55]. Fan et al. [56] reported that a single plant has the potential to generate substantial acylsugar diversification. Their findings concur with our findings as a range of sugars, such as trehalose, betaine, glucose, sucrose, and maltose, were detected in our study, as shown in Figure 3 and Figure 4.

Trehalose is a non-reducing disaccharide of glucose that stabilizes biological structures and macromolecules as proteins and membrane lipids during water deficits [57]. Recent studies by Nemadodzi et al. and Nemadodzi and Managa, [25,41] revealed that trehalose and betaine function as growth-promoting metabolites. A study by Kamau et al. [23] on *S. nigrum.* berries showed a steady increase in the glucose detected compared to other varieties. According to Fan et al. [49], sucrose and glucose are the predominant acylsugars in Solanaceae crops produced in type I/IV trichomes. They are implicated in insect defense and possibly desiccation tolerance, as reported by Feng et al. [58]. In another study by Halford et al. [59], sucrose, glucose, and maltose were reported to be the most abundant sugars among the world’s major crop plants. Advanced evidence has shown that the above-mentioned sugars not only play a role in plant growth but also act as a sugar-sensing system that initiates changes in gene expression [60]. The detection of glucose with a concentration (mM) of 3.6166 and a maximum (mM) of 3.1394 in the *S. nigrum* cultivated in the greenhouse could be due to the lack of natural sunlight, which is readily available in open-field-grown *S. nigrum*.

In addition, Kaplan and Guy [61] revealed that maltose can protect proteins, membranes, and the photosynthetic electron transport chain at physiologically relevant concentrations under heat and freezing stress. Furthermore, a study by Ritte and Raschke [62] confirmed that maltose was a major product of the catabolism of starch in guard cells. As reported by Verslues et al. [63], plants frequently encounter various environmental stresses during their development processes and have evolved a series of adaptive changes at both transcriptional and post-transcriptional levels, leading to the re-configuration of regulatory networks to maintain homeostasis. From the current findings, it is assumed that trehalose, betaine, glucose, and sucrose are the growth-promoting metabolites of *S. nigrum*., irrespective of the growth condition; on the other hand, maltose is regarded as a stress-inhibiting metabolite.

Caffeine is one of the most popular purine alkaloids of plant origin, and its content depends on the species of the plant and the organ from which the raw material originated [64]. Caffeine has been found in more than 60 plants, such as *Camellia sinensis* L. (tea) [65], *Coffee arabica* L. (coffee) [66], and *Theobroma cacao* L. (cacao) [67], and recently in *A. graecizans* and *A. cruentus* (red amaranth) species [42]. In South Africa, the leaves—and sometimes the tender shoots—are the edible parts of *S. nigrum*. However, *S. nigrum* is well known for its distinct bitter taste such that traditional cooking methods include boiling for +/- 45 min until the leaves are tender. The bitter taste, which could be attributed to the presence of caffeine, is reduced by severely discarding the dark brown boiled water. To our knowledge, this is the first study to identify and report caffeine in the leaves of *S. nigrum*. A recent study by Nemadodzi and Managa [42] reported metabolites such as choline and alanine as flavor agents and defense mechanism metabolites, respectively.

Chlorogenate (caffeoylquinate) is a widespread phenolic ester in plants that can accumulate to high levels [27]. It is one of the metabolites that have value in the nutraceutical market, and it is found in so-called superfoods [67]. Chlorogenates have recognized human health benefits, such as antioxidant, antiviral, hepatoprotective, and antihypoglycemic properties [68], as well as anti-inflammatory [69], anticancer [70,71,72,73,74], antidiabetic [75], antihypertensive [76], and antineurodegenerative activities [77,78,79]. Furthermore, several studies on chlorogenates have been conducted on the *S*. *lycopersicum* fruit, reporting the presence of polyphenols that play a role in plant stress response [80,81,82,83,84]. To our knowledge, this is the first study to detect and identify chlorogenate in the leaves *S. nigrum*.

In the current study, 3-chlorotyrosine was detected and identified in the leaves of *S. nigrum*, as shown in Figure 3 and Figure 4. According to Domigan et al. Kettle [85,86], 3-chlorotyrosine is formed when hypochlorous acid (HOCI) reacts with tyrosine residues in protein. Hypochlorous acid is a strong oxidizing agent, destroying target organisms via oxidation of their cellular material [87]), and it has been shown to be effective in eliminating large populations of microorganisms and extending the shelf life of many foods, including meat, poultry, and fish and their by-products, as well as fruits and vegetables [88,89,90]. Conventionally, at post-harvest, *S. nigrum* is stored at a cool room temperature of 22 °C [21] to maintain its leaf-quality, chlorophyll content, and moisture retention to prevent de-generation for at least 5–7 days, which could be due to the presence of 3-chlorotyrosine, before the leaves transition to a yellow color and ultimately wilt. Bao Loan et al. [91] disagree with our findings, reporting that 3-chlorotyrosine was only produced and found in “ready to eat” (RTE) vegetables such as *Lactuca sativa* L., commonly known as lettuce. Several studies by Bergt et al., Buss et al., Dalle-Donne et al., Davies et al., and Robaszkiewicz et al. [92,93,94,95,96] agree with our findings that 3-chlorotyrosine serves as a useful biomarker of protein damage by myeloperoxidase; it has been observed in a variety of pathological processes, such as atherosclerosis, glomerulonephritis, cystic fibrosis, rheumatoid arthritis, asthma, and Alzheimer’s disease. Bao Loan et al. [87] reported that 3-chlorotyrosine can potentially be used as an indicator for the treatment of fish fillets with hypochlorite. It should be emphasized that this is the first study to identify and report 3-chlorotyrosine in the leaves of *S. nigrum*.

As mentioned above, in South Africa, *S. nigrum* is regarded as a “medicinal vegetable” that is consumed to cure several human ailments due to its healing properties, which could be attributed to the metabolites it possesses. Interestingly, traditionally, *S. nigrum* has been highly consumed by elders (both males and females) diagnosed with diabetes and high blood pressure in their quest to minimize and maintain their sugar level and blood pressure, which could be associated with chlorogenates (unpublished Indigenous knowledge). In addition, there is a “scientific myth” and “belief” that drinking boiled-bitter water from the cooked *S. nigrum* acts as a pain killer for people living with arthritis.

On the other hand, glycocholate is described as one of the very important bile salts in humans [97]. According to Mukidjam et al. [98], bile salts are the most natural detergents, and they are formed from highly acidic conjugated acids [99]. In a study by Favretto et al. [100], glycocholate was found to occupy the large internal cavity of the human liver without requiring major conformational rearrangement of the protein backbone, which led to increased stability, similar to that estimated for fatty acid-binding protein (oleate complex). Studies by Hermens et al., Merkus et al., and Morimoto et al. [101,102,103] reported that glycocholate slightly affected the nasal ciliary beat frequencies. From the results of the current study, 3-chlorotyrosine, chlorogenate, and glycocholate are regarded as medical metabolites, confirming *S. nigrum* to be a medicinal vegetable.

## 5. Conclusions

In most African countries, including South Africa, indigenous green leafy vegetables have been perceived as “food for the poor” by mostly high- and middle-income earners, which has led to their very low to non-existent consumption. However, the use of advanced scientific tools such as ^1^H NMR to determine the metabolomics profile of IGLVs previously associated with poverty continues to unearth and reveal important metabolite compounds that contain numerous human health benefits. Although *S. nigrum* may not be highly preferred due to its bitter taste, assumed to be associated with the presence of caffeine, it possesses medicinal properties from metabolites such as 3-chlorotyrosine, chlorogenate, and glycocholate. Including indigenous green leafy vegetables such as *S. nigrum* in the human diet will ensure that humans gain medicinal properties known to prevent major “chronic and communicable” diseases such as heart disease, diabetes, cancer, inflammation, and viral diseases, thereby enhancing the quality of life. Since numerous plant metabolites need to be studied and identified on the IGLVs, metabolomics studies such as this are recommended to help in the construction of a plant metabolites database and a record of their medicinal benefits. Future studies still need to evaluate the potential use of *S. nigrum* fruits as a nutritious feed supplement for livestock, which might provide nutrients like vitamins and minerals essential to supporting animal health, productivity, food security, and nutrition, especially in regions where this valuable crop thrives well in the local climate and soil conditions.

## Figures and Tables

**Table 1:** The goodness of fit and predictability values of the PCA.

Component	R2X (cum)	Q2 (cum)
1	0.47	0.641
2	0.291	0.323

**Table 2:** The goodness of fit and predictability values of the OPLS-DA.

Component	R2X (cum)	Q2 (cum)
1	0.252	0.977
2	0.683	

**Table 3:** Secondary metabolites detected in the leaves of *S*. *nigrum* grown in open fields.

Metabolites	Structural Fragment (Methyl Group)	NMR Region	Chenomx 9.0	Human Metabolome Data Base
Alanine (1)	(CH_3_)	1.45 1.46	1.5 3.8	1.47 3.77
Glycocholate (2)	C (4); C (14)	1.0 1.5 1.6 1.7 1.8	0.7 0.9 1.0 1.3 1.6 1.7 1.8 1.9 2.0 2.1 2.2 2.4 3.5 3.7 3.9 4.0 7.9	N/A
Choline (3)	S-adenosyl-L-methionine	3.2	3.2 3.5 4.1	N/A
Betaine (4)	(CH_3_)_3_N^+^CH_2_COO	3.3 3.9	3.3 3.9	3.25 3.89 [43]
Caffeine (5)	Methyl isocyanate CH_3_NCO	3.3 3.5 3.9	3.3 3.5 3.9 7.9	3.22 3.34 4.04 8.49 [44]
Galactose (6)	3-O-methylated or 4-O-methylated	4.6	3.5 3.7 3.8 3.9 4.0 4.1 4.6 5.3	N/A
Trehalose (7)	C16–19 C21–25 C24–28	5.19	3.4 3.6 3.8 3.9 5.2	3.42 3.49 3.63 3.75 3.81 3.84 3.85 4.12 [45]
Maltose (8)	C1 C4	5.38	3.3 3.4 3.6 3.7 3.8 3.9 4.0 4.6 5.2 5.4	N/A
Sucrose (9)	6-&6-	5.4	3.4 3.6 3.7 3.8 3.9 4.0 4.2 5.4	N/A
Chlorogenate (10)	[M-H]	7.0	2.0 2.1 2.2 3.9 4.3 5.3 6.9 7.1 7.2 7.6	N/A
3-Chlorotyrosine (11)		7.0	3.0 3.2 3.9 7.0 7.1 7.3	2.82 2.83 2.85 2.86 3.06 3.07 3.09 3.10 3.97 3.98 3.99 6.82 6.84 7.16 7.18 7.62

**Table 4:** Secondary metabolites detected in the leaves of *S*. *nigrum* grown in the greenhouse.

Metabolites	Structural Fragment (Methyl Group)	NMR Region	Chenomx 9.0	Human Metabolome Data Base
Alanine (1)	(CH_3_)	1.45 1.46	1.5 3.8	1.47 3.77
Glycocholate (2)	C (4); C (14)	1.0 1.5 1.6 1.7 1.8	0.7 0.9 1.0 1.3 1.6 1.7 1.8 1.9 2.0 2.1 2.2 2.4 3.5 3.7 3.9 4.0 7.9	N/A
Choline (3)	S-adenosyl-L-methionine	3.2	3.2 3.5 4.1	N/A
Betaine (4)	(CH_3_)_3_N^+^CH_2_COO	3.3 3.9	3.3 3.9	3.25 3.89 [43]
Caffeine (5)	Methyl isocyanate CH_3_NCO	3.3 3.5 3.9	3.3 3.5 3.9 7.9	3.22 3.34 4.04 8.49 [44]
Galactose (6)	3-O-methylated or 4-O-methylated	4.6	3.5 3.7 3.8 3.9 4.0 4.1 4.6 5.3	N/A
Trehalose (7)	C16–19 C21–25 C24–28	5.19	3.4 3.6 3.8 3.9 5.2	3.42 3.49 3.63 3.75 3.81 3.84 3.85 4.12 [45]
Maltose (8)	C1 C4	5.38	3.3 3.4 3.6 3.7 3.8 3.9 4.0 4.6 5.2 5.4	N/A
Sucrose (9)	6-&6-	5.4	3.4 3.6 3.7 3.8 3.9 4.0 4.2 5.4	N/A
Chlorogenate (10)	[M-H]	7.0	2.0 2.1 2.2 3.9 4.3 5.3 6.9 7.1 7.2 7.6	N/A
3- Chlorotyrosine (11)		7.0	3.0 3.2 3.9 7.0 7.1 7.3	2.82 2.83 2.85 2.86 3.06 3.07 3.09 3.10 3.97 3.98 3.99 6.82 6.84 7.16 7.18 7.62
Glucose (12)	3-O-methyl-D-glucose	3.4 4.0	3.2 3.4 3.5 3.7 3.8 3.9 4.7 5.2	

## Data Availability

The data presented in this study are available on request from the corresponding author.
